# IL-33 deficiency causes persistent inflammation and severe neurodegeneration in retinal detachment

**DOI:** 10.1186/s12974-019-1625-y

**Published:** 2019-12-03

**Authors:** Josy Augustine, Sofia Pavlou, Imran Ali, Kevin Harkin, Ema Ozaki, Matthew Campbell, Alan W. Stitt, Heping Xu, Mei Chen

**Affiliations:** 1 Wellcome-Wolfson Institute for Experimental Medicine, School of Medicine, Dentistry & Biomedical Science, Queen’s University Belfast, Belfast, Northern Ireland, UK; 2 Smurfit Institute of Genetics, Trinity College Dublin, Dublin, Ireland

**Keywords:** Retinal detachment, IL-33, Neurodegeneration, Müller cells, Macrophages

## Abstract

**Background:**

Interleukin-33 (IL-33) belongs to the IL-1 cytokine family and resides in the nuclei of various cell types. In the neural retina, IL-33 is predominately expressed in Müller cells although its role in health and disease is ill-defined. Müller cell gliosis is a critical response during the acute phase of retinal detachment (RD), and in this study, we investigated if IL-33 was modulatory in the inflammatory and neurodegenerative pathology which is characteristic of this important clinical condition.

**Methods:**

RD was induced by subretinal injection of sodium hyaluronate into C57BL/6 J (WT) and *IL-33*^*−/−*^ mice and confirmed by fundus imaging and optical coherence tomography (OCT). The expression of inflammatory cytokines, complement components and growth factors was examined by RT-PCR. Retinal neurodegeneration, Müller cell activation and immune cell infiltration were assessed using immunohistochemistry. The expression of inflammatory cytokines in primary Müller cells and bone marrow-derived macrophages (BM-DMs) was assessed by RT-PCR and Cytometric Bead Array.

**Results:**

RD persisted for at least 28 days after the injection of sodium hyaluronate, accompanied by significant cone photoreceptor degeneration. The mRNA levels of CCL2, C1ra, C1s, IL-18, IL-1β, TNFα, IL-33 and glial fibrillary acidic protein (GFAP) were significantly increased at day 1 post-RD, reduced gradually and, with the exception of GFAP and C1ra, returned to the basal levels by day 28 in WT mice. In *IL-33*^*−/−*^ mice, RD induced an exacerbated inflammatory response with significantly higher levels of CCL2, IL-1β and GFAP when compared to WT. Sustained GFAP activation and immune cell infiltration was detected at day 28 post-RD in *IL-33*^*−/−*^ mice. Electroretinography revealed a lower A-wave amplitude at day 28 post-RD in *IL-33*^*−/−*^ mice compared to that in WT RD mice. *IL-33*^*−/−*^ mice subjected to RD also had significantly more severe cone photoreceptor degeneration compared to WT counterparts. Surprisingly, Müller cells from *IL-33*^*−/−*^ mice expressed significantly lower levels of CCL2 and IL-6 compared with those from WT mice, particularly under hypoxic conditions, whereas *IL-33*^*−/−*^ bone marrow-derived macrophages expressed higher levels of inducible nitric oxide synthase, TNFα, IL-1β and CCL2 after LPS + IFNγ stimulation compared to WT macrophages.

**Conclusion:**

IL-33 deficiency enhanced retinal degeneration and gliosis following RD which was related to sustained subretinal inflammation from infiltrating macrophages. IL-33 may provide a previously unrecognised protective response by negatively regulating macrophage activation following retinal detachment.

## Background

Retinal detachment (RD) is a sight-threatening condition that can occur spontaneously or as a complication of trauma, inflammation and in diseases such as age-related macular degeneration and proliferative diabetic retinopathy. Retinal Müller cells are central to the pathophysiology of RD, being involved in gliosis, initiation of inflammatory cascades and/or proliferative responses [1]. Reactive microglia and macrophages are also prevalent in the RD retina where they play a key role in neurodegeneration and photoreceptor depletion [2]. Microglia may also have a protective role as depletion of microglia resulted in increased photoreceptor death in experimental RD [3]. Dysregulated Müller cell activation can lead to or exacerbate this pathology, with evidence suggesting that there is direct communication with professional immune cells through the CD40-ATP-P_2_X_7_ pathway [4]. Recent studies have shown that interleukin (IL)-33, a nuclei-binding cytokine, is expressed predominately in Müller cells in the neuroretina [5] although its role in retinal glial pathophysiology remains ill-defined.

IL-33 belongs to the IL-1 cytokine family which includes IL-1β and IL-18. Unlike other members of this family, IL-33 is constitutively expressed at high levels in the nuclei of many cell types including endothelial, glial and some immune cells [6]. Under healthy conditions, IL-33 participates in maintaining physiological function of IL-33-expressing cells by regulating gene expression as a nuclear protein [6, 7]. Nuclear IL-33 negatively affects gene transcription in an intracrine fashion by sequestration of NF-kB leading to the repression of gene expression to dampen pro-inflammatory pathways [6, 7]. Nuclear IL-33 can also function as an “alarmin” once it is released from damaged or dead cells [6, 7]. When occurring extracellularly, IL-33 binds to a heterodimeric receptor complex consisting of ST2 and the IL-1R accessory protein (IL-1RAP) which induces signalling cascades through the Toll/interleukin-1 receptor (TIR) domain [6, 8]. Activation of the IL-33 receptor induces a Th2-type immunity [9], which can be pro-inflammatory (such as IL-1β, TNFα, IL-4, IL-6, and CCL2) [10] or anti-inflammatory (e.g., IL-10) [11].

Previously, we have shown that IL-33 can suppress retinal inflammation in a mouse model of autoimmune uveoretinitis [12], while Theodoropoulou et al. reported that this cytokine can protect the retina from pathogenic angiogenesis through reducing the activation and migration of choroidal endothelia and fibroblasts [13]. In contrast to studies showing IL-33-mediated protection against retinal pathology, Xi and colleagues have reported that IL-33-expressing-Müller cells can promote inflammation and contribute to the pathogenesis of age-related macular degeneration [14]. Therefore, the precise role of IL-33 in inflammation-mediated, acute-phase retinal neurodegeneration warrants further investigation.

In the current study, we assessed the role of IL-33 in inflammatory and neurodegenerative pathology arising from RD. We have found that the expression of IL-33 is increased in the detached retina, which is accompanied by reactive glial activation. Genetic deletion of IL-33 resulted in sustained retinal inflammation, Müller glial activation and a more severe photoreceptor degeneration in RD.

## Methods

### Animals and animal procedures

*IL-33*^*−/−*^ mice [15] were obtained from RIKEN Center for Life Science Technologies (Japan, http://www.clst.riken.jp/arg/mutant%20mice%20list.html, Accession No CDB0631K). This colony was on the C57BL/6 N background and carried the rd8 mutation. The colony was cross-bred with C57BL/6 J (WT) mice to eliminate the rd8 mutation in the animal facility in Trinity College Dublin before transferred to Queen’s University Belfast. C57BL/6 J (WT) and *IL-33*^*−/−*^ mice were maintained in the Biological Service Unit (BSU) at Queen’s University Belfast and had free access to food and water. In vivo procedures were approved by the UK Home Office Animals (Scientific Procedures) Act 1986 and local Animal Welfare and Ethical Review Board (AWERB) and were in compliance of the Association for Research in Vision and Ophthalmology (ARVO) Statement for the use of Animals in Ophthalmology and Vision Research.

RD was induced by subretinal injection of sodium hyaluronate (Alcon, TX, USA) into 3–4-month-old mice [16, 17]. Briefly, the pupils were dilated with 1% atropine sulphate and 2.5% phenylephrine hydrochloride (Chauvin, Essex, UK) and the animals were anesthetised with isoflurane (Merial Animal Health Ltd., Essex, UK). Viscotears Liquid Gel (Novartis Pharmaceuticals Ltd., Surrey, UK) was then applied on the eyes to keep them moist, and the vitreous cavity was visualised under a surgical microscope with the aid of applying a cover slip on top of the cornea. Then a 33 G needle connected to a microsyringe/dispenser (Hamilton Bonaduz AG, Bonaduz, Switzerland) was inserted into the subretinal space and 2 μl of sodium hyaluronate was gently injected to detach the neurosensory retina from the underlying retinal pigment epithelia (RPE). The eyes were collected at different time points as indicated in the “Results” section.

Ganzfeld electroretinography (ERG) was performed on mice at day 28 post-RD using a Diagnosys Espion system (Diagnosys Technologies, MA, USA) in compliance with the manufacturer’s guidelines. Mice were dark-adapted overnight, and the procedures were conducted under dim-red light (< 1 lx). Mice were anaesthetised with ketamine (Vetoquinol UK Ltd., Buckingham, UK) and Rompun (Bayer Health Care, Kiel, Germany) and the pupils dilated and moisturised as described above. Scotopic ERG was recorded via mouse corneal ERG electrodes in response to a single white light flash produced by the Diagnosys Espion ERG system. For each animal, 8 light intensities ranging from 0.008 to 25 cd s/m^2^ were applied. A-wave and B-wave amplitudes were measured using the Espion analysis software (Diagnosys Technologies, USA).

Spectral domain optical coherence tomography (SD-OCT) was performed using the Spectralis Heidelberg OCT system (Heidelberg Engineering, Heidelberg, Germany), according to manufacturers’ instructions. Mice were anesthetised and the pupils dilated and moisturised as described above. OCT images (30° field of view) were collected from mice at day 28 post-RD.

Fundus images of the retina were acquired using a Micron IV mouse fundus camera (Phoenix Research Laboratories, CA, USA) according to manufacturers’ instructions. Mice were anesthetised and the pupils were dilated and moisturised as described above. Fundus photographs were collected from mice at day 7 and day 28 post-RD.

### Ocular histology study

The eyes were collected at different time points following RD and fixed in 2% paraformaldehyde (Sigma-Aldrich, Dorset, UK) for 2 h at room temperature post-enucleation. The eyes were then washed with PBS and placed in 10%, 20% and 30% sucrose (Sigma-Aldrich, USA) before embedment in OCT (optimal cutting temperature) compound and cryo-sectioned using Leica CM 1900 cryostat (Leica Microsystems, Milton Keynes, UK) at 16 μm thickness. H & E and immunofluorescent staining was performed following published protocols [18, 19] and antibodies used were listed in Table 1. A negative control, without the primary antibody, was carried out in each staining.

**Table 1:** Antibodies used for immunohistochemistry and western blot

Antibody	Dilution	Company	Catalogue number
CD68	1:300	BioRad	MCA1957
Cone-arrestin	1:1000	Millipore	AB15282
GFAP	1:200	DAKO	Z0334
GS	1:2000	Sigma	G2781
IL-33	1:50	R & D	AF3626
Donkey anti-goat IgG Alexa Fluor 488	1:300	Jackson Immunoresearch	705–545-147
Donkey anti-rabbit IgG Alexa Fluor 488	1:300	Jackson Immunoresearch	711–585-152
Goat anti-rat IgG Alexa Fluor 594	1:300	Invitrogen	A-11007

Images were acquired using a Nikon C1 Eclipse TE200-U (Nikon, UK) or an Olympus IX51 inverted fluorescent microscope (Olympus, UK) with the same settings for each antibody. Images were processed using Fiji software (provided in the public domain, https://imagej.net/Fiji/). Briefly, the number of CD68^+^ immune cells, DAPI-labelled nuclei and cone-arrestin^+^ photoreceptor cells were counted using a multi-point tool in Fiji. The number of rod cells was calculated by subtracting the number of cone-arrestin^+^ cells from the total DAPI^+^ cells in the ONL [20]. Synaptophysin^+^ areas in the OPL and IPL areas were measured using a free-hand selection tool in Fiji. GFAP quantification was achieved by counting GFAP-positive fibres within the INL layer and post the INL layer of the retina. The data were presented as mean ± standard error of the mean (SEM), normalised to 100 μm of retinal length.

### Real-time qRT-PCR

Total RNA was extracted using RNeasy Mini Kit (Qiagen, Hilden, Germany) and the same amount of RNA was transcribed into cDNA using SuperScript II Reverse Transcriptase Kit (ThermoFisher Scientific, Winsford, UK), following the manufacturer’s instructions. The following validated TaqMan probes were purchased from Roche (Basel, Switzerland): IL-1β (310471), IL-33 (316824), IL-6 (300699), IL-18 (301115), CCL2 (310467), vascular endothelial growth factor A (VEGFA, 314944), glial fibrillary acidic protein (GFAP, 311752), transforming growth factor-β (TGFβ, 317139), tumour necrosis factor-α (TNFα, 317484), β-actin (307903) and 18S (307906). SYBR Green gene specific primers were designed using the National Center for Biotechnology Information (NCBI) Primer Blast and synthesised by Integrated DNA Technologies (Leuven, Belgium). Primer sequences were listed in Table 2. Quantitative RT-PCR was performed using a Roche LightCycler 480. Relative gene expression was calculated using the comparative Ct method (2^−ΔCt^) with data normalised to β-actin or 18S.

**Table 2:** SYBR Green Primer sequences

Name of Gene	Forward primer	Reverse primer
C1ra	GCCATGCCCAGGTGCCAAGATCAA	TGGCTGGCTGCCCTCTGATG
GS	TGCCTGCCCAGTGGGAATT	TATTGGAAGGGTTCGTCGCC
C1s	TGGACAGTGGAGCAACTCCGGT	GGTGGGTACTCCACAGGCTGGAA
18S	AGGGGAGAGCGGGTAAGAGA	GGACAGGACTAGGCGGAACA

### Cell culture

Primary Müller cell (PMC) cultures were established from the dissected retina of postnatal day 7 pups [21]. Phenotypes of Müller cell were confirmed (Additional file1: Figure S1). 2.5 × 10^4^ cells were seeded into 12-well plates and cultured in standard cell culture incubator or hypoxic chamber (1% O_2_) for 24 h. After which, supernatants were collected and cells were lysed for RNA extraction.

Bone marrow-derived macrophages (BM-DMs) were cultured from 8- to 12-week mice using a protocol established in the lab previously [22]. Briefly, bone marrow cells were flushed out from mouse tibias and femurs with DMEM and then filtered through 100-μm cell strainer (BD, Oxford, UK). Red blood cells were lysed with lysis buffer (0.75% NH4Cl, 0.02% Tris–HCl, pH 7.2) on ice for 15 min. Bone marrow cells were then cultured in DMEM supplemented with 10% FBS and 20% L929 conditioned medium for 7 days, re-plated and stimulated with LPS (lipopolysaccharides, 50 ng/ml, Sigma-Aldrich, Missouri, USA) and IFN-γ (interferon-γ, 100 ng/ml, Bio-Techne, Minnesota, USA) [22]. Supernatants were collected after 24 h, and cells were lysed for RNA extraction.

### Cytokine quantification

Supernatants were measured for IL-6, TNFα and CCL2 using Cytometric Bead Array (CBA) Flex sets following the manufacturer’s guidelines (BD Biosciences, Wokingham, UK). BD FACSCanto II was used to acquire the samples, and data were analysed using the FCAP Array™ Software (BD Biosciences, UK). Nitric oxide (NO) was measured using the Griess Assay according to the manufacturer’s instructions (Promega, Madison, Wisconsin, USA).

### Statistical analysis

Data were presented as mean ± SEM. Statistical analysis was performed using Prism 6 (GraphPad Software, CA, USA). Normality and equal variance were tested prior to use unpaired student’s t-test (with or without Welch’s correction), one-way analysis of variance (ANOVA) or two-way ANOVA with appropriate post hoc tests. *P* < 0.05 was considered statistically significant.

## Results

### Persistent retinal detachment induces photoreceptor degeneration

**Fig. 1:**
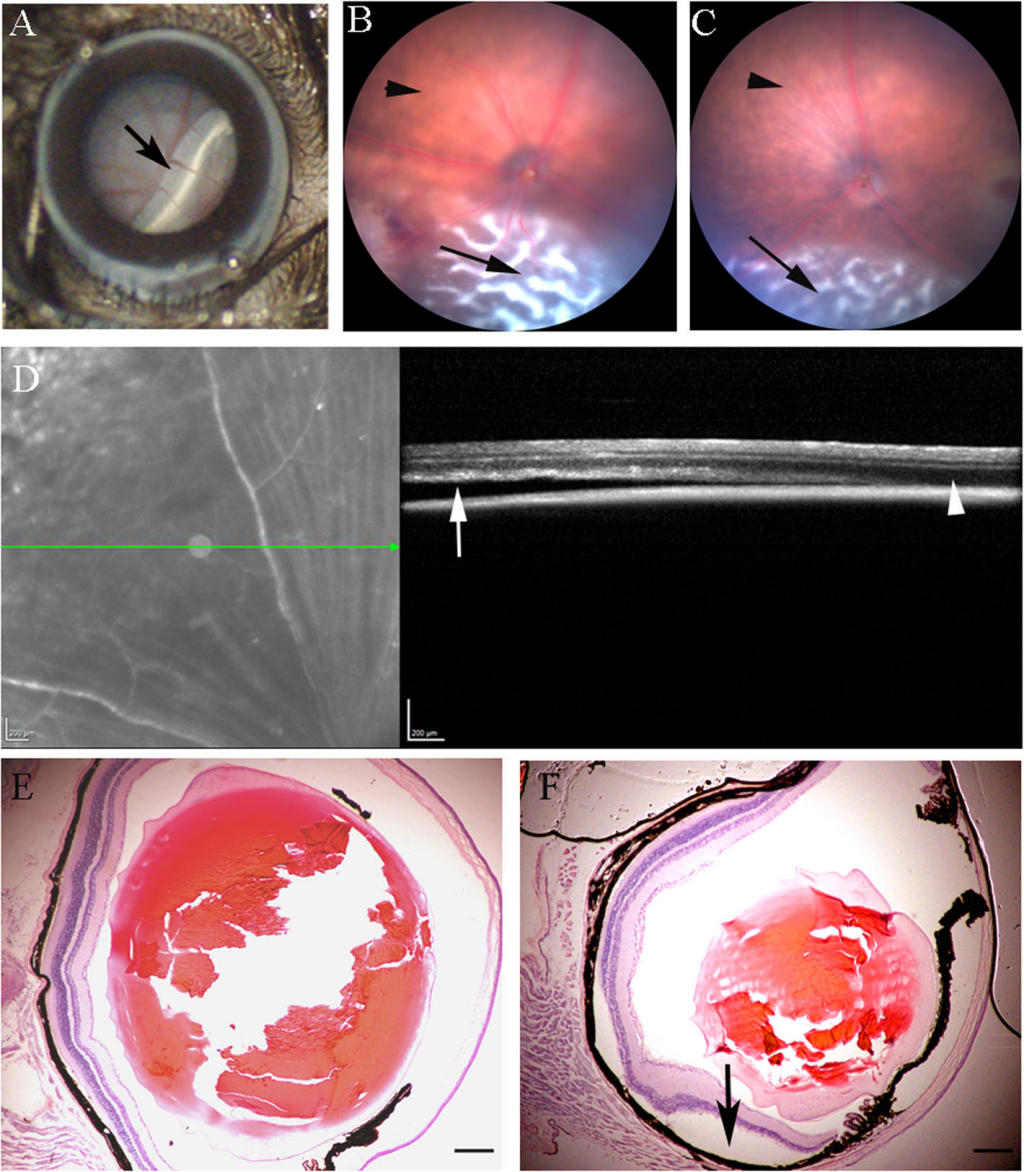
Retinal detachment (RD) and photoreceptor degeneration followed the injection of sodium hyaluronate. Two microliters of sodium hyaluronate was injected into the subretinal space to detach the neuroretina from RPE. **a** Representative photograph showing retinal detachment immediately following the injection of sodium hyaluronate. Arrow, detached retina. **b**, **c** Fundus image showing retinal damage at day 7 (**b**) and day 28 (**c**) post-RD. Arrow, detached area; arrowhead, non-detached area. **d** SD-OCT showing detached (arrow) and non-detached area (arrowhead) in the same eye at day 28 post-RD. Photoreceptor layer is thinner in the detached area (arrow). **e**, **f** Histology showing the intact photoreceptor cells and segments in normal ocular section (**e**) and detached retina (**f**, arrow, photoreceptor and segment loss) at day 28 post-RD. Scale bar, 100 μm

In the RD model, the injection of sodium hyaluronate resulted in detachment of the neuroretina from the underlying RPE layer (Fig. 1a, arrow). The retina remained detached during the entire course of this study (28 days) (Fig. 1b, c). Retinal detachment and photoreceptor degeneration were confirmed by SD-OCT (Fig. 1d) and histology (H & E staining) (Fig. 1e, f).

### IL-33 and inflammatory gene expression in the detached retina

**Fig. 2:**
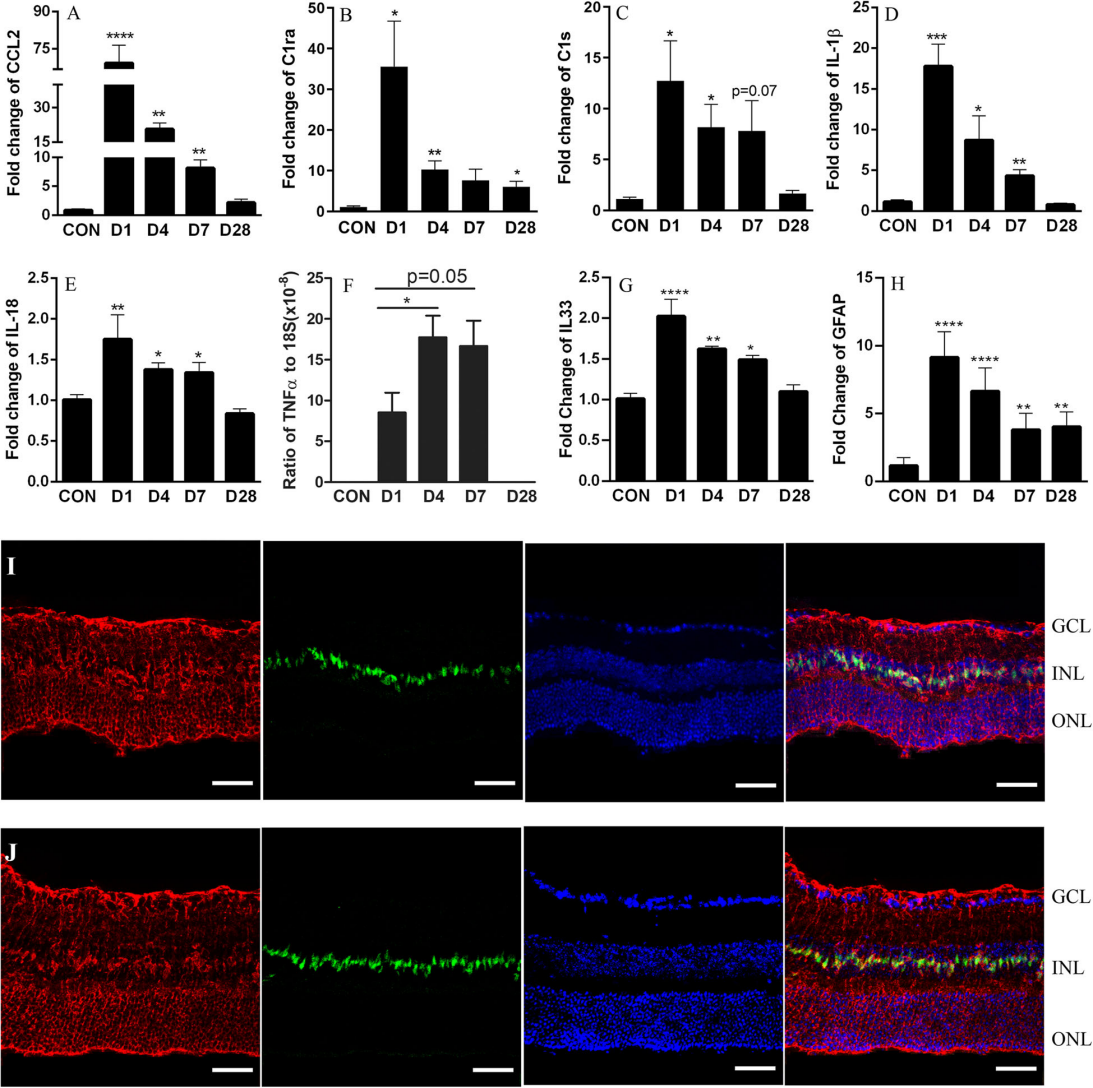
RD results in an acute inflammatory response in WT retina. At different days following RD, the retinas were collected and processed for RT-PCR analysis of inflammatory genes in WT mice (**a**–**f**). The mRNA expression of CCL2 (**a**), C1ra (**b**), C1s (**c**), IL-1β (**d**) and IL-18 (**e**) at days 1, 4, 7, and 28 post-RD. **f** The expression level of TNFα peaked at day 4 and remained at the peak at day 7 and returned back to the basal level at day 28 post-RD. **g** The expression level of IL-33 peaked at day 1, then decreased towards the basal level at day 28 post-RD. **h** The expression level of GFAP peaked at day 1 and reduced gradually. At day 28 post-RD, the expression level of GFAP was maintained at a higher level than control. Data presented as mean ± SEM, *n* = 6 per group, one-way ANOVA followed by Dunnett’s multiple comparisons test, *P < 0.05, **P < 0.01, ***P < 0.001. **i**, **j** Representative images of retinal sections stained for glutamine synthetase (red, marker for Müller cells), IL-33 (green) and DAPI (blue) in **i** detached retina (day 28 post-RD) and **j** normal retina. IL-33 was localised in the nuclei of Müller cells, and no significant IL-33 signal (green) could be observed out of Müller cells in detached retina. Scale bar, 50 μm

Quantitative RT-PCR revealed a significant inflammatory response following RD. In WT RD mice, the expressions of CCL2 (Fig. 2a), complement components C1ra (Fig. 2b) and C1s (Fig. 2c), cytokines IL-1β (Fig. 2d), IL-18 (Fig. 2e), IL-33 (Fig. 2g) and GFAP (Fig. 2h) peaked in the detached retina at day 1. TNFα (Fig. 2f) was not detectable in normal retinal tissues. It became detectable post-RD and peaked at day 4 (Fig. 2f). Expression levels were then gradually reduced and returned to basal levels at day 28 (Fig. 2a, c–g), except C1ra and GFAP, which were maintained at significantly higher levels than that of the controls (Fig. 2b, h). IL-33 was predominantly located in the nuclei of Müller cells (Fig. 2i, j) in both detached (Fig. 2i) and normal retina (Fig. 2j). The data suggest that RD induced an acute retinal inflammation that lasted for at least 7 days. The inflammatory response resolved by 28 days after RD.

### IL-33 deficiency results in sustained inflammation following retinal detachment

**Fig. 3:**
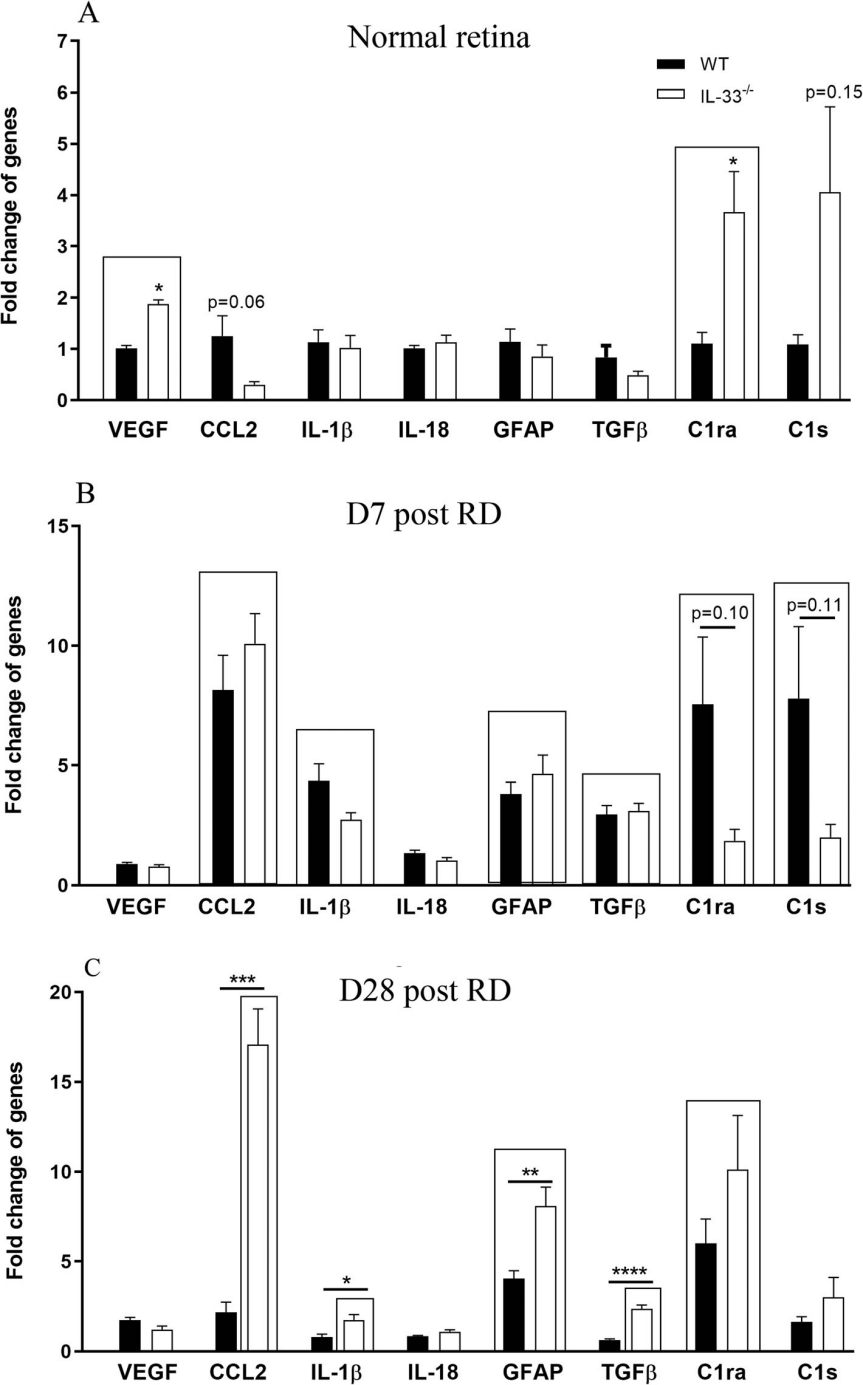
Deletion of IL-33 results in sustained retinal inflammation and gliosis post-RD. **a** Expression levels in normal WT and *IL-33*^*−/−*^ mice. *IL-33*^*−/−*^ retina expressed significantly higher levels of VEGF and C1ra. CCL2, IL-1β, IL-18, GFAP, TGFβ and C1s were at similar levels. **b** Expression levels at day 7 post-RD. Gene expression levels from day 7 were compared with control retina from the same strain. Upregulated genes to that of RD control were identified in boxes. Expression levels were increased at the similar level in both strains. **c** Expression levels of retina at day 28 post-RD. Gene expression levels from day 28 were compared with that of control retina from the same strain. Upregulated genes to that of RD control were identified in boxes. Most mRNA levels returned to basal levels at day 28; however, CCL2, IL1β, GFAP and TGFβ in *IL-33*^*−/−*^ retina were higher than in WT. **a**–**c** *n* = 6, Student’s *t* test, *P < 0.05, **P < 0.01, ***P < 0.001

**Fig. 4:**
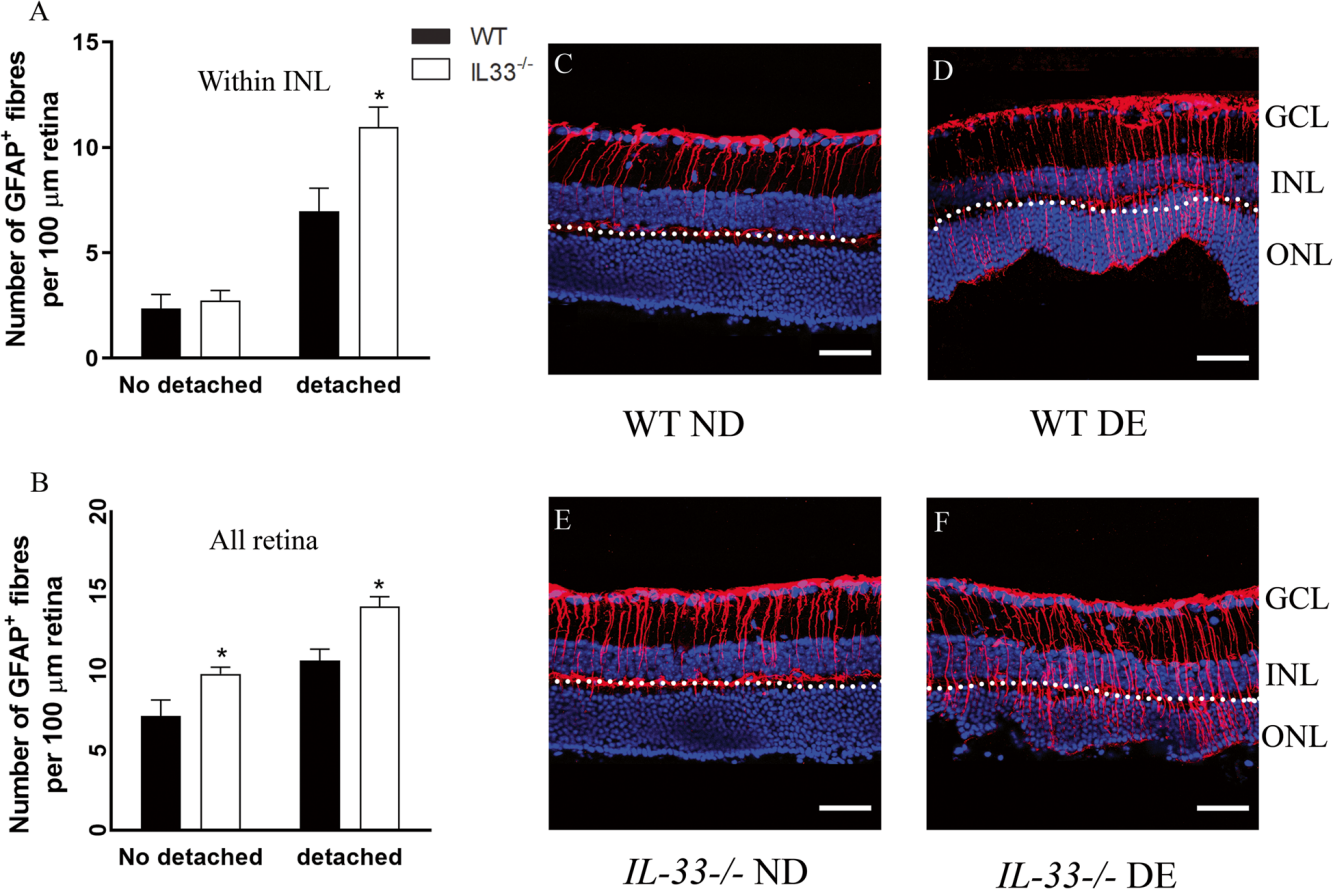
GFAP expression in the retina from WT and *IL-33*^*−/−*^ mice day 28 post-RD. The number of GFAP^+^ fibres retained within the GCL and INL layers (**a**) and across the whole retina (**b**) in the detached and non-detached regions from the same retina. Data presented as mean ± SEM. *n* = 5 mice, 3–6 sections per retina, two-way ANOVA followed by Bonferroni’s multiple comparisons test, *P < 0.05. **c**–**f** Representative images of day 28 post-RD showing GFAP (red) and DAPI (blue) in **c** WT non-detached, **d** WT detached, **e** *IL-33*^*−/−*^ non-detached and **f** *IL-33*^*−/−*^ detached regions. GCL ganglion cell layer, INL inner nuclear layer, ONL outer nuclear layer, ND non-detached region, DE detached region. Scale bar, 50 μm

To understand the role of IL-33 in RD-induced retinal inflammation, we compared inflammatory gene expression in the retinas of WT mice and *IL-33*^*−/−*^ mice. Under normal non-diseased conditions, TNFα and IL-6 (data not shown) were almost undetectable in the retinas of both strains although *IL-33*^*−/−*^ mice had significantly higher levels of VEGF and C1ra compared with the retina from WT mice (Fig. 3a). The expression levels of other genes including CCL2, IL-1β, IL-18, GFAP, TGFβ and C1s did not significantly differ between both strains (Fig. 3a). Seven days following RD, the expression of CCL2, IL-1β, GFAP, TGFβ, C1ra and C1s were increased in both WT and *IL-33*^*−/−*^ mice and the upregulated ratios to non-detached control retinas were at the similar levels (Fig. 3b). The expression levels of VEGF and IL-18 was not affected by RD in both WT and *IL-33*^*−/−*^ mice (Fig. 3b). TNFα was detectable in both strains, but IL-6 remained undetectable in all RD retina (data not shown). At day 28 post-RD, the expression of inflammatory genes returned to basal level in WT mice (Fig. 3c), except GFAP and C1ra. However, the expression levels of CCL2, IL-1β and TGFβ from *IL-33*^*−/−*^ RD mice were still significantly upregulated in comparison to non-detached control retinas (Fig. 3c). GFAP remained upregulated in both WT and *IL-33*^*−/−*^ retinas at day 28 post-RD and was significantly higher in *IL-33*^*−/−*^ mice than those in WT retina (Fig. 3c). The fold change of GFAP mRNA at day 28 (8.09±1.05) was higher than that at day 7 (4.64±0.49) (Fig. 3b, c). Immunofluorescent staining on ocular sections also demonstrated increased GFAP reactivity in both regions of detached and non-detached retina in *IL-33*^*−/−*^ mice compared to that in WT mice at day 28 post-RD (Fig. 4a–f).

**Fig. 5:**
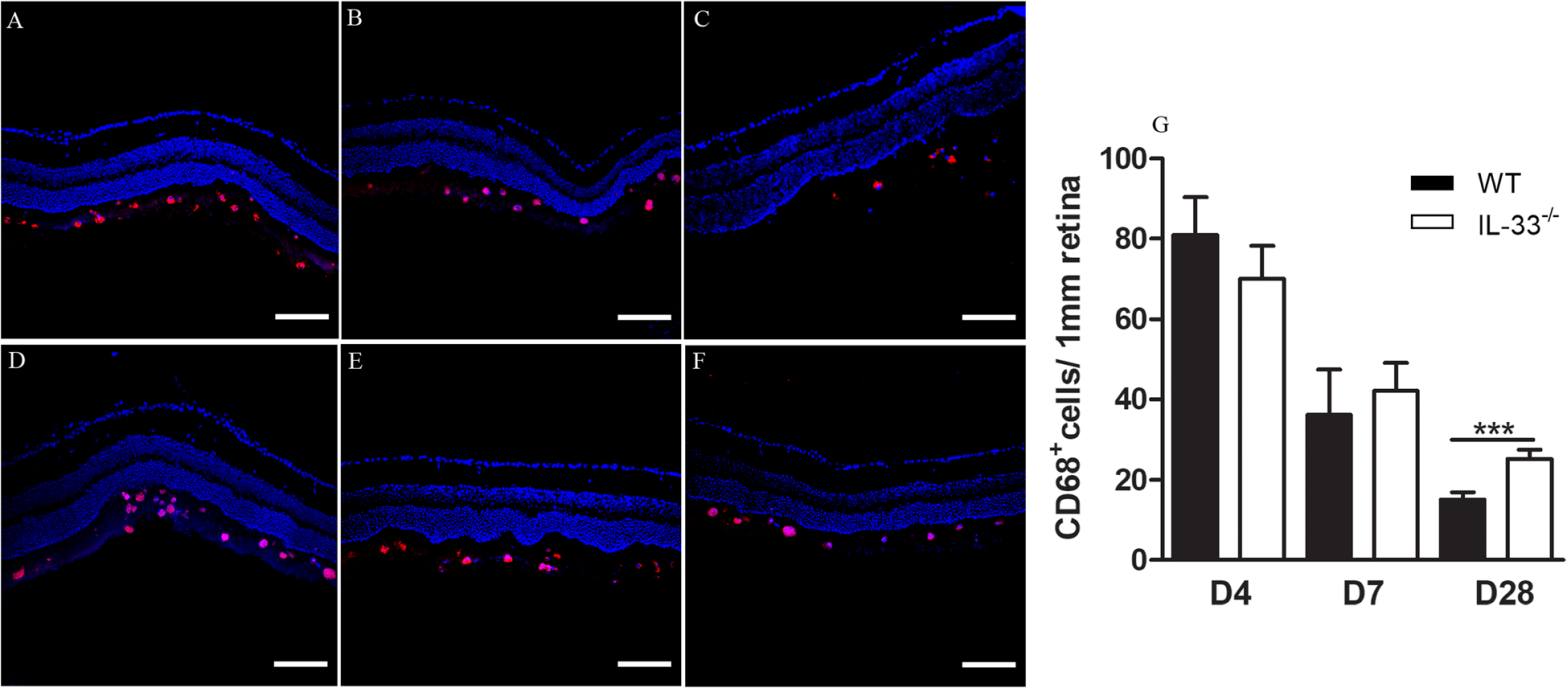
Deletion of IL-33 results in increased immune cell infiltration following detachment. **a**–**f** Representative images of WT and *IL-33*^*−/−*^ retinal sections stained for CD68 (red) and DAPI (blue) in **a** WT day 4, **b** WT day 7, **c** WT day 28, **d** *IL-33*^*−/−*^ day 4, **e** *IL-33*^*−/−*^ day 7 and **f** *IL-33*^*−/−*^ day 28 post-RD. **g** The number of CD68^+^ cells in the subretinal spaces of WT and *IL-33*^*−/−*^ eyes at day 4, day 7 and day 28 post-RD. Data were presented as mean ± SEM. *n* = 5 mice, 3–6 sections per retina, Student’s *t* test were used to compare the cell number between two groups at the same time point. ***P < 0.001. Scale bar, 100 μm

CD68^+^ immune cells were detected in the subretinal space/outer retinal layer spaces in the detached areas of RD mice. Similar numbers of CD68^+^ cells were detected at day 4 (Fig. 5a, d, g) and day 7 (Fig. 5b, e, g) in WT and *IL-33*^*−/−*^ mice. However, a significantly higher number of CD68^+^ cells were detected in the subretinal/outer retinal layer spaces in *IL-33*^*−/−*^ mice at day 28 compared to that in WT mice (Fig. 5c, f, g).

Taken together, our data suggest that deletion of IL-33 resulted in persistent retinal gliosis and subretinal inflammation following retinal detachment.

### IL-33 deficiency results in more severe neuron degeneration following retinal detachment

**Fig. 6:**
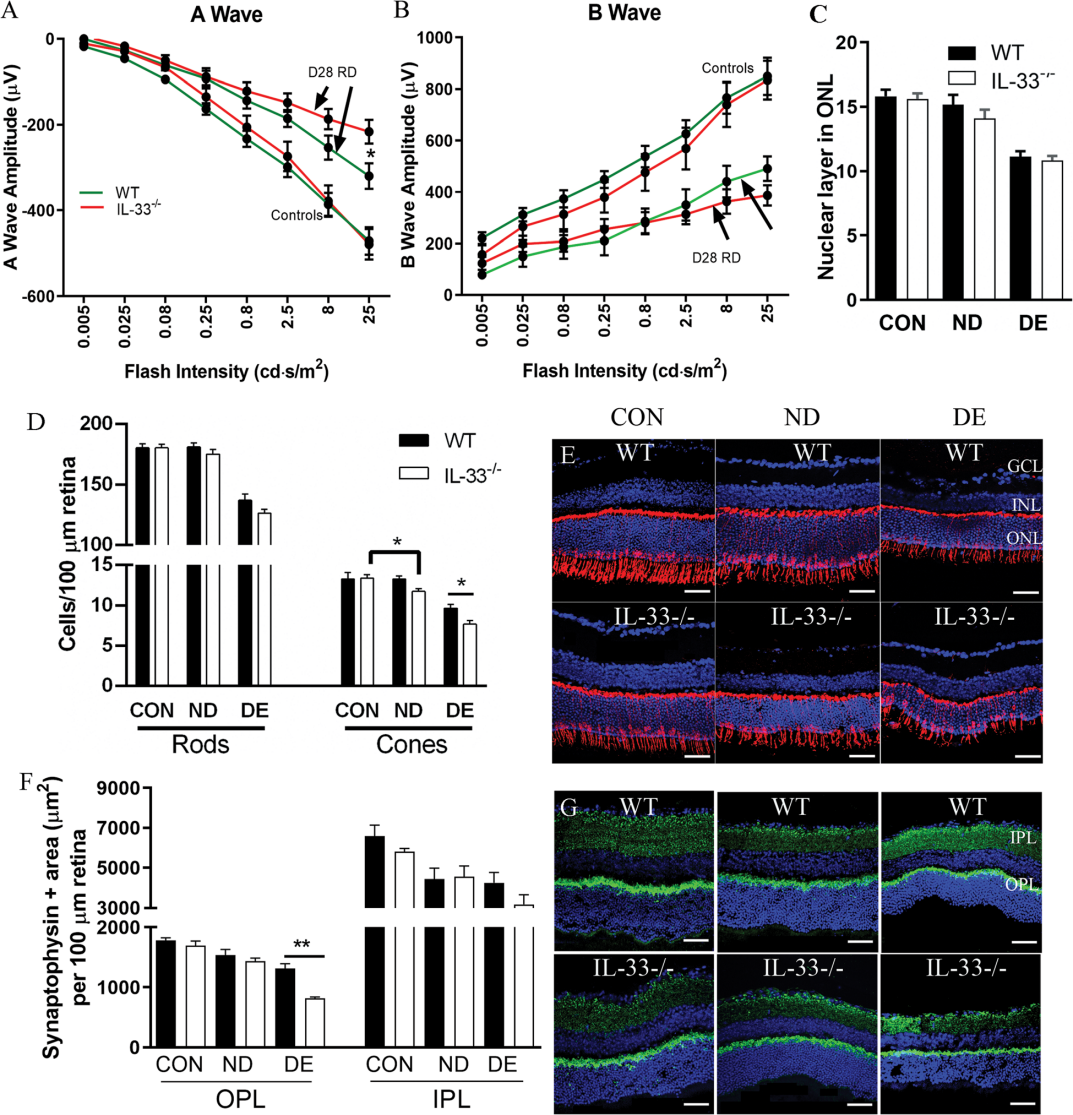
Deletion of IL-33 results in more severe neuron degeneration following RD. **a–b** Scotopic ERGs of WT and *IL-33*^*−/−*^ at day 28 post-RD. Line graph of A-wave amplitudes (**a**) and B-wave amplitudes (**b**) at flash intensities ranging from 0.008 to 25 cd s/m^2^ were quantified. Data were presented as mean ± SEM, *n* = 5–12 eyes per experimental condition; arrow, day 28 post-RD. Student’s *t* test was used to compare the amplitudes between WT and *IL-33*^*-/-*^ at the same flash intensity. *P < 0.05. **c–e** Photoreceptor degeneration in WT and *IL-33*^*−/−*^ retina at day 28 post-RD was investigated using immunohistochemistry of cone-arrestin. **c** The number of nuclear layer of the ONL in normal retina, non-detached area and detached area of RD retina. **d** The number of rod cells, cone-arrestin^+^ cells in normal retina, non-detached area and detached area of RD retina. **e** Representative images of WT and *IL-33*^*−/−*^ retinal sections stained for cone-arrestin (red) and DAPI (blue). **f–g** Synaptic integrity in WT and *IL-33*^*−/−*^ retina at day 28 post-RD was investigated using immunohistochemistry of synaptophysin. **f** The area of synaptophysin in OPL and IPL in normal retina and detached and non-detached regions of RD retina. **g** Representative images of WT and *IL-33*^*−/−*^ retinal sections stained for synaptophysin (green) and DAPI (blue). GCL ganglion cell layer, INL inner nuclear layer, ONL outer nuclear layer, OPL outer plexiform layer, IPL inner plexiform layer, CON control retina, ND non-detached region of retina having RD, DE detached region of retina having RD. Data presented as mean ± SEM. *n* = 5 mice, 3–6 sections per retina. Student’s *t* test was used to compare the means between WT and *IL-33*^*-/-*^ at the same condition. *P < 0.05, **P < 0.01. Scale bar, 50 μm

To understand the impact of persistent gliosis and subretinal inflammation to photoreceptor degeneration in *IL-33*^*−/−*^ RD mice, visual function was examined by ERG at day 28 post-RD. There was no difference in the amplitude of A- and B-waves between normal *IL-33*^*−/−*^ and WT mice (Fig. 6a, b). Retinal detachment resulted in significant reductions in the amplitudes of both A- and B-waves in both strains; however, the reduction in A-wave was more severe in *IL-33*^*−/−*^ RD mice compared to that in WT RD mice (Fig. 6a, b).

Reduction of A-wave suggested photoreceptor cells degeneration. The number of photoreceptor nuclear layer (Fig. 6c), the number of cone arrestin^+^ cells (Fig. 6d), and the number of rod photoreceptor cells (Fig. 6d) were quantified from images stained with cone arrestin and DAPI (Fig. 6e). The number of photoreceptor nuclear layers and the number of cone and rod photoreceptor cells were similar in control WT and *IL-33*^*−/−*^ mice (Fig. 6c–e). There is no significant reduction in nuclear layers and rod photoreceptor cells at the non-detached area of the detached eyes compared to the control eyes in both WT and *IL-33*^*−/−*^ mice (Fig. 6c–e). The number of cone-arrestin^+^ cell at the non-detached area of the detached eyes remained unchanged compared to that of the non-detached control eyes in WT mice but was significantly reduced in *IL-33*^*−/−*^ mice (Fig. 6c–e). Photoreceptor nuclear layers in the detached areas were significantly thinner than in normal eyes and non-detached area in the detached eye in both WT and *IL-33*^*−/−*^ mice; however, there was no difference between WT and *IL-33*^*−/−*^ mice. The number of rod photoreceptor cells and cone-arrestin^+^ cells in the detached area was significantly reduced in both WT and *IL-33*^*−/−*^ mice, and the reduction in cone-arrestin^+^ cells was more severe in *IL-33*^*−/−*^ mice compared to that in WT mice (Fig. 6d–e).

Synaptic vesicles were identified using synaptophysin immunostaining. Areas of synaptophysin in the outer plexiform layer (OPL) and inner plexiform layer (IPL) were quantified as previously described [20]. The synaptophysin^+^ areas were at the comparable levels in both the OPL and IPL in non-detached control WT and *IL-33*^*−/−*^ mice. RD resulted in significantly reduced synaptophysin-positive regions in the OPL and IPL in both detached area and non-detached area at day 28 post-RD in both *IL-33*^*−/−*^ and WT mice (Fig. 6f, g). In the IPL, the reduction levels in the detached area or non-detached area were similar between the two strains of mice, whereas the reduction was more severe in the OPL of detached areas in *IL-33*^*−/−*^ mice compared to that in WT mice (Fig. 6f, g). This data shows that RD-induced photoreceptor and synaptophysin loss was more severe in *IL-33*^*−/−*^ mice compared to that in WT mice.

### IL-33 deficiency does not induce inflammatory phenotype in primary Müller cells

**Fig. 7:**
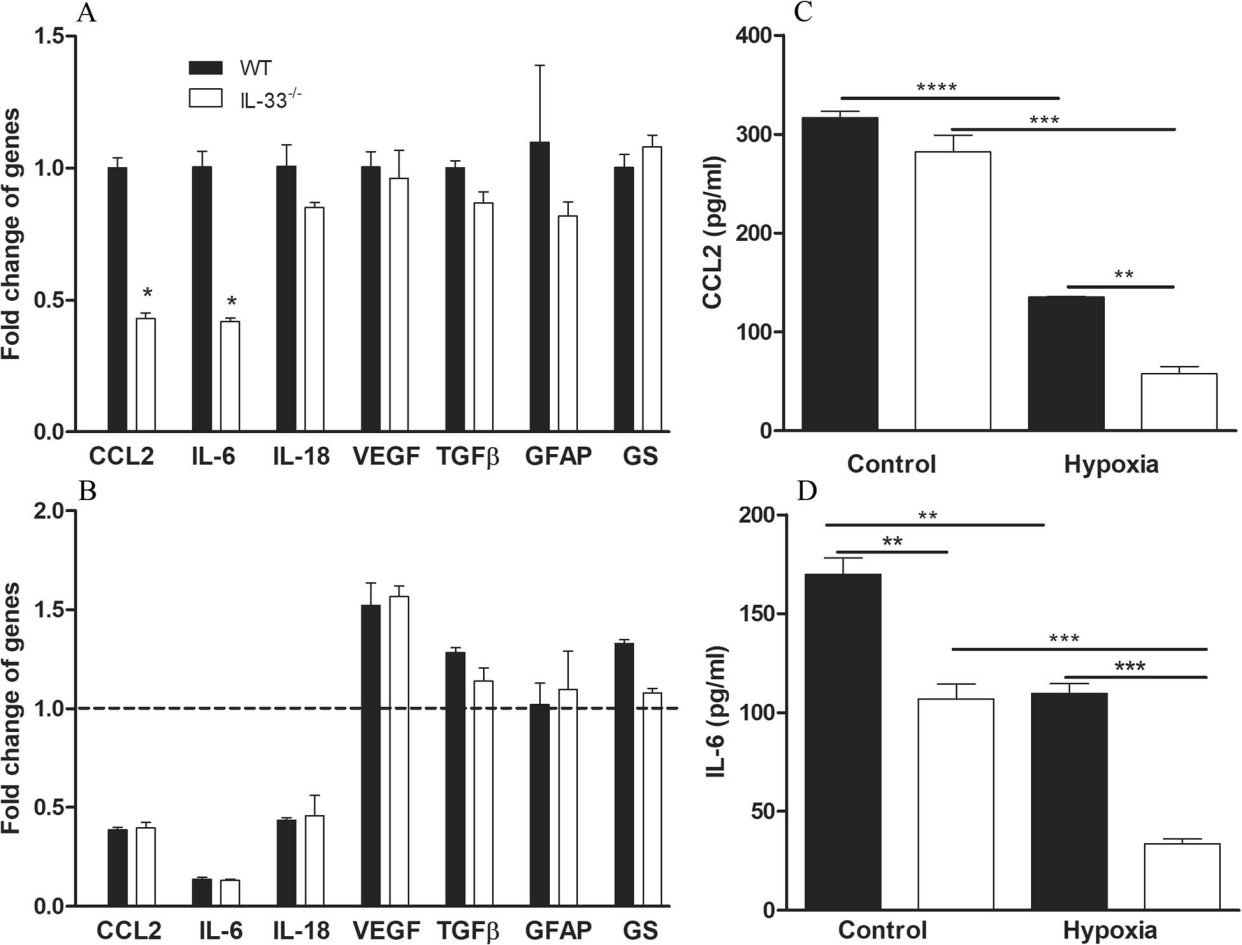
Primary Müller cells from WT and *IL-33*^*−/−*^ mice present similar inflammatory response. **a** mRNA levels of CCL2, IL-6, IL-18, VEGF, TGFβ, GFAP, GS in WT and *IL-33*^*−/−*^ PMC cultured in standard incubators. Expression levels of CCL2 and IL-6 was lower in *IL-33*^*−/−*^ PMC, whereas other genes were at similar levels. **b** mRNA level of PMCs cultured in 1% hypoxia for 24 h. Expression level was compared to PMC from the same strain. Fold changes above the dotted line suggested increased response in hypoxia (VEGF); fold changes below the dotted line suggested decreased response in hypoxia (CCL2, IL-6 and IL-18). **c**, **d** Cytokine concentration in culture supernatants were measured by CBA. Hypoxia reduced the secretion of CCL2 and IL-6 in both WT and *IL-33*^*−/−*^ PMCs. Data presented as mean ± SEM, *n* = 3. **a**, **b** Student’s *t* test. **c**, **d** Two-way ANOVA followed by Tukey’s multiple comparisons. *n* = 6, *P < 0.05, **P < 0.01, ***P < 0.001. CBA cytometric beads array

Since IL-33 is predominately expressed by Müller cells in the retina, we then investigated inflammatory cytokine expression in PMCs from WT and *IL-33*^*−/−*^ mice. Under normal culture conditions, PMCs from *IL-33*^*−/−*^ mice expressed significantly lower levels of CCL2 and IL-6 compared to the cells from WT mice (Fig. 7a). The expression levels of IL-18, VEGF, TGFβ, GFAP and glutamine synthetase (GS) were comparable between WT and *IL-33*^*−/−*^ cells (Fig. 7a). The mRNA expression of TNFα and IL-1β were undetectable in both PMCs (data not shown).

Under hypoxic conditions (1% O_2_), the expression of VEGF was significantly increased in PMCs isolated from WT and *IL-33*^*−/−*^ mice (Fig. 7b), whereas the expression of CCL2, IL-6 and IL-18 was significantly decreased in both PMCs compared to that in normal control culture conditions (Fig. 7b). Other genes, including TGFβ, GFAP and GS were not altered by hypoxia in both types of cells (Fig. 7b). Cytokines released into the culture media were selectively measured using CBA. Hypoxia significantly reduced the production of CCL2 in both WT and *IL-33*^*−/−*^ Müller cells (Fig. 7c), and the reduction were more pronounced in *IL-33*^*−/−*^ PMCs (Fig. 7c). IL-6 production was lower in *IL-33*^*−/−*^ PMC under control conditions (Fig. 7d), hypoxia significantly reduced the production of IL-6 in both cell types (Fig. 7d) and the reduction was more significant in* IL-33*^*−/−*^ PMCs (Fig. 7d). Our data suggest that IL-33 deletion suppresses a pro-inflammatory phenotype in Müller cells and reduces CCL2 and IL-6 production under control and hypoxic conditions.

### Macrophages from* IL-33*^*−/−*^ mice express higher levels of inflammatory cytokines

**Fig. 8:**
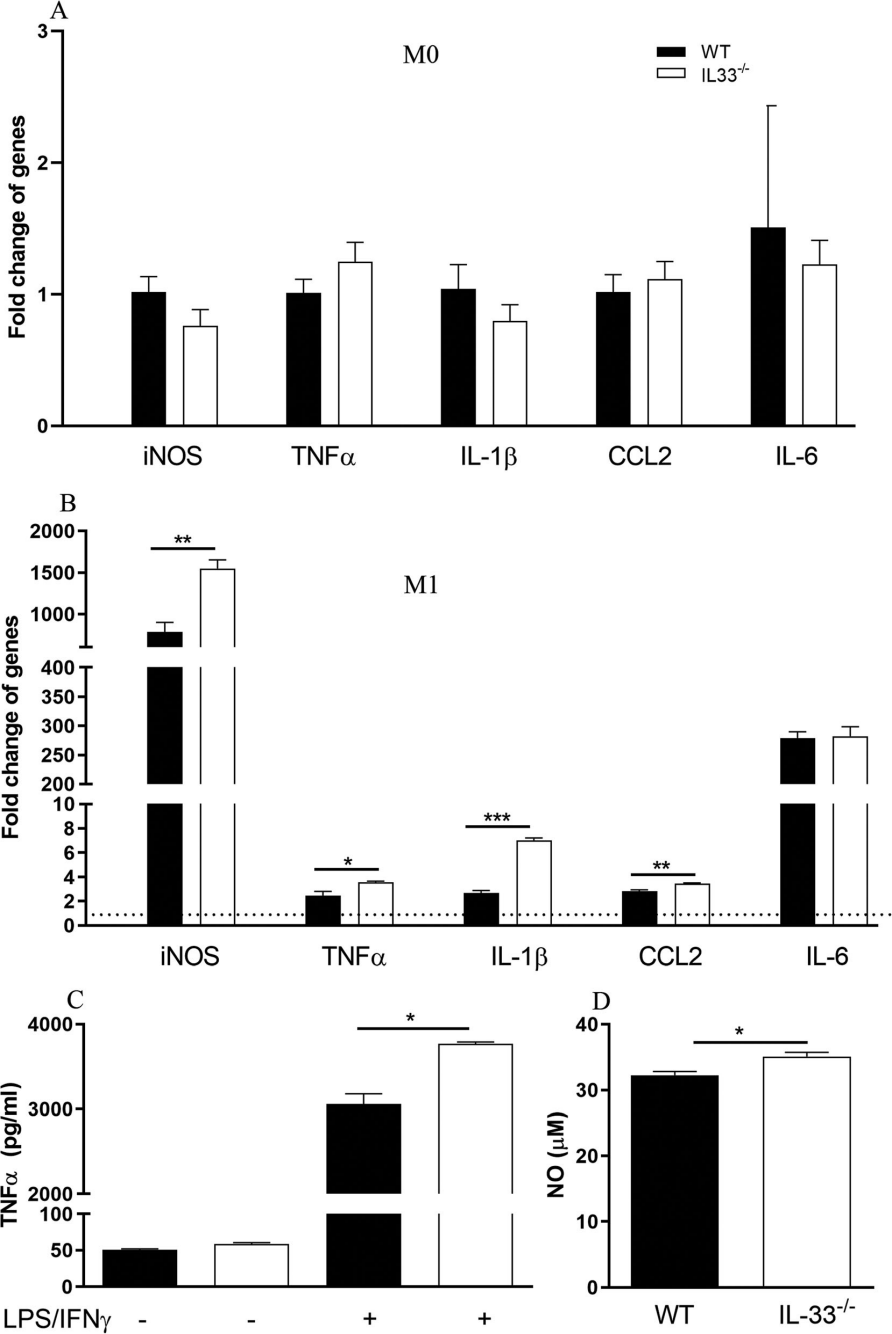
Bone marrow-derived macrophages from *IL-33*^*−/−*^ mice are more pro-inflammatory after classic stimulation (LPS/IFNγ). **a**, **b** mRNA expression of inflammatory genes including iNOS, TNFα, IL1-β, CCL2 and IL-6 before (**a: M0**) or after (**b: M1**) LPS + INFγ treated BM-DMs cultured from WT and *IL-33*^*−/−*^ mice. Y-axis showing the fold changes of mRNA expression level compared to non-stimulated BM-DM. Dot line levels fold change at 1. RNA levels were upregulated after stimulation for each cell type. The increased levels of iNOS, TNFα, IL-18 and CCL2 of *IL-33*^*−/−*^ BMDMs were significantly higher than those of WT BM-DMs. **c** Secretion of TNFα was higher in *IL-33*^*−/−*^ BM-DM than in WT. **d** The concentration of NO released into the culture medium from *IL-33*^*−/−*^ BM-DM was higher than those in WT BMDM. NO nitric oxide. Data presented as mean ± SEM, *n* = 3, Student’s *t* test, *P < 0.05, **P < 0.01, ***P < 0.001

BM-DMs showed similar levels of pro-inflammatory gene expressions before the treatment of LPS + IFNγ (Fig. 8a). Following LPS + INFγ stimulation, BM-DMs from *IL-33*^*−/−*^ mice showed higher levels of pro-inflammatory gene expressions, including CCL2, IL-1β, TNFα and inducible nitric oxide synthase (iNOS), than those from WT BM-DMs (Fig. 8b), the expression levels of IL-6 were similar between WT and *IL-33*^*−/−*^ cells (Fig. 8b). TNFα and nitric oxide (NO) in the supernatants from *IL-33*^*−/−*^ macrophages were also higher than those from WT macrophages (Fig. 8c and d).

## Discussion

In this study, we show that IL-33 is upregulated in Müller cells following experimental RD and this was accompanied by a sustained gliosis and subretinal inflammation. We also showed that *IL-33*^*−/−*^ mice developed more severe retinal degeneration following retinal detachment.

Early study on adult cat has shown that RD-induced apoptotic photoreceptor death (TUNEL^+^ cells) occurs at the acute (i.e., day 1 and day 3 after RD) but not the chronic (day 28 after RD) stage of the disease [23]. This observation was further confirmed in mouse and rat models of RD by Hisatomi and colleagues [24], where the authors detected photoreceptor death as early as 12 h after RD, which peaked at day 3 and then gradually diminished [24]. Hisatomi et al. also found that the ONL thickness continued to decrease over 28 days after RD [24], suggesting that an apoptotic-independent photoreceptor degeneration exists at the late stages of RD. Multiple pathways are known to be involved in the RD-induced photoreceptor death, including the caspase pathway (through apoptosis or inflammasome activation), autophagy, mitochondrial dysfunction, inflammation and gliosis [25]. GFAP is a marker of gliosis and is “non-specifically” increased in reactive glia. In the normal retina, GFAP is expressed in astrocytes and the end-feet of Müller cells at the optic nerve/ganglion cell layer (GCL). The expression of GFAP in Müller cell is upregulated upon activation and can be detected across the entire cell body. Previous studies have shown that deletion of GFAP or vimentin can prevent RD-induced gliosis and rescue photoreceptor degeneration, which highlights the critical role of Müller cells play in modulating the acute response to retinal damage [26]. IL-33 is expressed predominately in Müller cells in the retina, and we found that its expression was further increased in the acute phase of RD. In the current study, RD resulted in more severe gliosis in *IL-33*^*−/−*^ mice, accompanied by a more severe photoreceptor degeneration as evidenced by a significantly lower number of cone-arrestin^+^ cells, reduced synaptic vesicles in OPL and reduced A-wave response in ERG. The physiological function of gliosis is not fully understood although it has been linked to both protective and detrimental roles [1]. In our study, primary Müller cell cultures from WT and *IL-33*^*−/−*^ mice express comparable levels of GFAP under control and hypoxic conditions; GFAP gliosis in WT and *IL-33*^*−/−*^ mice was comparable at the acute phase of RD (within 7 days). But the *IL-33*^*−/−*^ mice has more severe gliosis at the chronic stages of RD (i.e., at day 28), suggesting that IL-33 may be critically involved in RD-induced inflammatory response at the late stages of the disease.

Although IL-33 is considered as an alarmin when released from damaged cells, it also has important immune regulatory roles. In the central nervous system (CNS), the glia-derived IL-33 is known to orchestrate tissue immune response and promote recovery after injury [27]. IL-33 is expressed by oligodendrocytes and is released upon CNS injury. The extracellular IL-33 then binds to the receptor ST2 on microglia and astrocytes, leading to chemokine (e.g., CCL2) production and subsequent recruitment of circulating immune cells [27]. Mice lacking IL-33 have impaired recovery after CNS injury due to less myeloid cell, in particular, reparative M2 macrophage infiltration at the injury site [27]. IL-33 can also promote resolving of inflammation and recovery from acute colitis by inducing miR-320 to stimulate epithelial restitution and repair [28]. Interestingly, the IL-33/ST2 pathway may also participate in a pro-inflammatory response and contribute to CNS damage. Cao and colleagues reported that LPS-induced neuroinflammation is reduced in *IL-33*^*−/−*^ mice and microglia from *IL-33*^*−/−*^ mice produce significantly lower levels of inflammatory mediators such as TNF-α, IL-6, IL-1β and CCL2 after LPS treatment [29]. The opposite roles of IL-33 in CNS injury and LPS-induced neuroinflammation suggests that IL-33 may function differently under different disease conditions.

We have shown previously that IL-33 can suppress retinal inflammation in a mouse model of autoimmune uveoretinitis through induction of type 2 cytokines and M2 macrophages [12]. Theodoropoulou et al. reported that IL-33 has anti-angiogenic roles in the mouse model of laser-induced choroidal neovascularisation through the reduction of choroidal endothelial and fibroblast activation and migration [13]. However, Xi and colleagues have found that IL-33^+^ Müller cells can promote retinal inflammation in retinal injury and during age-related macular degeneration [14]. The authors found that IL-33 was released by Müller cells after light exposure, which then induced CCL2, IL-6 and IL-1β expression through autocrine activation of Müller cells [14] and promoted immune cell infiltration and retinal degeneration [14]. In this study, we found that in vitro cultured Müller cells from *IL-33*^*−/−*^ mice produced lower levels of CCL2 and IL-6 compared to cells from WT mice, whereas the expression of TNFα, IL-1β, IL-10 and VEGF did not differ between the two types of cells. The results suggest that IL-33 may positively regulate CCL2 and IL-6 expression in Müller cells.

Seven days after retinal detachment, retinal inflammatory gene expression, CD68^+^ immune cell infiltration and GFAP expression were comparable between WT and *IL-33*^*−/−*^ mice, suggesting that IL-33 depletion did not affect RD-mediated defence response at the acute stages of retinal injury. RD-mediated retinal inflammatory response resolved almost completely in WT mice but persisted in *IL-33*^*−/−*^ mice at day 28. Our results suggest that IL-33 may play an important role in the resolution of inflammation in this model, which is in line with a previous study in the CNS [27].

Exactly how IL-33 participates in the resolution of retinal inflammation in our model system remains unknown. After the retina is detached from the RPE, damaged neurons release a large amount of damage-associated molecular patterns (DAMPs) that induce acute inflammation characterised by pro-inflammatory cytokine expression and macrophage (M1) infiltration. The resolution of inflammation may be initiated when the DAMPs are removed. Anti-inflammatory, tissue repair M2 macrophages are known to play an important role in resolving inflammation. Although macrophages are highly plastic, M1 macrophages are unable to re-differentiate into M2 macrophages due to irreversible damage to mitochondrial electron transport chain by nitric oxide during inflammatory activation [30]. Therefore, the anti-inflammatory macrophages must be recruited to the detached retina at the late stage of disease. The persistent inflammation in the *IL-33*^*−/−*^ RD retina may be related to the delayed clearance of inflammatory M1 macrophages or the lack of recruitment of or inability to induce anti-inflammatory M2 macrophages.

## Conclusion

In this study, we have demonstrated that RD resulted in persistent gliosis, sustained subretinal inflammation, immune cell infiltration and severe photoreceptor degeneration in *IL-33*^*−/−*^ mice. IL-33 may positively regulate CCL2 and IL-6 expression in Müller cells, but negatively regulate inflammatory macrophage activation. Based on the present data, the IL-33 pathway warrants further scrutiny as a possible therapeutic avenue in inflammatory and degenerative retinal diseases.

## Supplementary information

**Additionalfile1: Figure S1.** Immunofluorescent staining was conducted to confirm the phenotypes of primary Müller cells from WT and* IL-33*^*−/−*^ retinas. IL-33 (green) was positive in WT PMC, but not *IL-33*^*−/−*^ PMC. Both WT and *IL-33*^*−/−*^ PMCs are positive for common Müller cell markers including GS (red), GFAP (red), SMA (smooth muscle actin alpha, red), Kir4.1 (green), and Aquaporin 4 (red). DAPI is in blue*.* Scale bar: 25 μm. (TIF 254 kb)

## Data Availability

The datasets used during the current study are available from the corresponding author on reasonable request.

## Abbreviations

BM-DM: Bone marrow-derived macrophages
CCL2: Chemokine (C-C motif) ligand 2
ERG: Electroretinography
GCL: Ganglion cell layer
GFAP: Glial fibrillary acidic protein
GS: Glutamine synthetase
IL: Interleukin
INL: Inner nuclear layer
ONL: Outer nuclear layer
PMC: Primary Müller cell
RD: Retinal detachment
TNFα: Tumour necrosis factor-alpha
VEGFA: Vascular endothelial growth factor A
