# Supplementary figures and images for: IL-33 deficiency causes persistent inflammation and severe neurodegeneration in retinal detachment
Source: J Neuroinflammation. 2019 Dec 3;16:251. doi: 10.1186/s12974-019-1625-y (PMC6889479; doi:10.1186/s12974-019-1625-y)

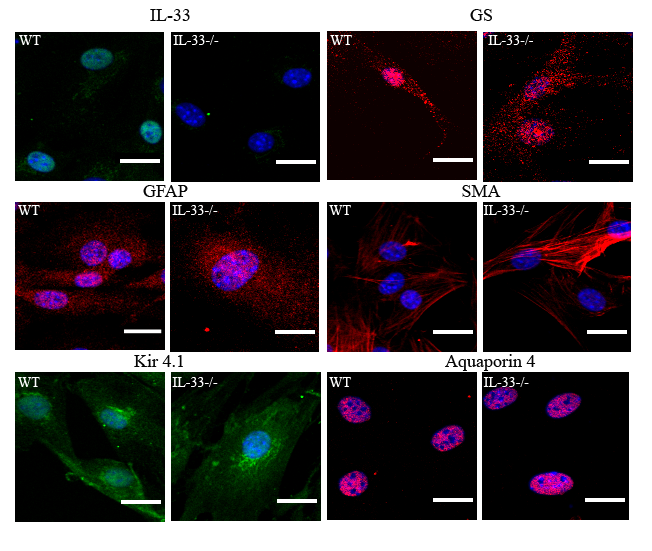

Supplement: Supplementary file 1 — Additionalfile1: Figure S1. Immunofluorescent staining was conducted to confirm the phenotypes of primary Müller cells from WT and IL-33−/− retinas. IL-33 (green) was positive in WT PMC, but not IL-33−/− PMC. Both WT and IL-33−/− PMCs are positive for common Müller cell markers including GS (red), GFAP (red), SMA (smooth muscle actin alpha, red), Kir4.1 (green), and Aquaporin 4 (red). DAPI is in blue. Scale bar: 25 μm. (TIF 254 kb) [file 12974_2019_1625_MOESM1_ESM.tif]
